# Repetitive transcranial magnetic stimulation in the treatment of resistant depression: changes of specific neurotransmitter precursor amino acids

**DOI:** 10.1007/s00702-021-02363-7

**Published:** 2021-07-09

**Authors:** F. Leblhuber, S. Geisler, D. Ehrlich, K. Steiner, G. Reibnegger, Dietmar Fuchs, K. Kurz

**Affiliations:** 1 Department of Gerontology, Kepler University Clinic, Linz, Austria; 2 Institute of Biological Chemistry, Innsbruck Medical University, Innrain 80, Room M04-313, 6020 BiocenterInnsbruck, Austria; 3 Division of Physiological Chemistry, Otto-Loewi Research Center, Graz Medical University, Graz, Austria; 4 Department of Internal Medicine, Innsbruck Medical University, Innsbruck, Austria

**Keywords:** Treatment-resistant depression, Transcranial magnetic stimulation, Phenylalanine, Tyrosine, Phenylalanine hydroxylase

## Abstract

Repetitive transcranial magnetic stimulation (rTMS) for treatment-resistant major depression offers an alternative therapy, since more than every third patient is not responding to adequate antidepressive treatment. In this interventional study safety, symptom development and changes of serum concentrations of neurotransmitter precursor amino acids, of immune activation and inflammation markers, of brain-derived neurotrophic factor (BDNF), nitrite as well as of salivary amylase were measured before and after a frontal polar cortex stimulation using rTMS as add-on treatment in 38 patients with treatment-resistant depression. Out of these, 17 patients received sham stimulation as a control. Treatment was well tolerated: with the exception of one patient of the verum group, who described discomfort during the second treatment, no serious adverse effects were observed. Improvement of depression with a significant decrease in the HAMD-7 scale (*p* = 0.001) was found in patients treated with rTMS, but not in sham-treated patients. Furthermore, serum phenylalanine and tyrosine dropped significantly (*p* = 0.03 and *p* = 0.027, respectively) in rTMS-treated patients. The kynurenine to tryptophan ratio (Kyn/Trp) tended to decrease under rTMS (*p* = 0.07). In addition, associations between concentrations of BDNF and neopterin as well as serum nitrite levels were found in patients after rTMS treatment, which indicates an influence of immune regulatory circuits on BDNF levels. In the sham-treated patients, no changes of biomarker concentrations were observed. Results show that rTMS is effective in the treatment of resistant depression. rTMS appears to influence the enzyme phenylalanine hydroxylase, which plays a central role in the biosynthesis of neurotransmitter precursors tyrosine and dihydroxyphenylalanine (DOPA).

## Introduction

Depression represents a substantial social and economic burden (Simon 2003) as the second most prevalent disease after cardiovascular syndromes. The global population living with depression is estimated as to be 4.4% of the world’s population (Friedrich 2017). Treatment-resistant depression is common with up to 50–60% of patients without adequate response to antidepressant medication (Fava 2003).

Earlier studies described inflammation and oxidative stress in addition to monoamine deficiency as biological factors in mood and anxiety disorders and in neurodegeneration (Dantzer et al. 2002; Widner et al. 2002; Capuron et al. 2011; Lindqvist et al. 2017; Felger, 2018). Tetrahydrobiopterin (BH4), the cofactor required for the enzymatic conversion of monoamine precursors tryptophan and tyrosine via phenylalanine hydroxylase (PAH), and tryptophan hydroxylase and tyrosine hydroxylase, which are essential for the biosynthesis of neurotransmitters serotonin, dopamine and norepinephrine, all these are dysregulated in patients with depressive syndromes (Sperner-Unterweger et al. 2014; Qiu et al. 2015).

Th1-type cytokine interferon-γ does not only stimulate the production of BH4, but also induces the formation of reactive oxygen species (ROS) in macrophages at the same time (Nathan et al. 1983). ROS may destroy oxidation-labile BH4 and the loss of BH4 caused by oxidative stress then reduces the biosynthesis of catecholamines, thereby possibly causing neurotransmitter deficits in depression (Neurauter et al. 2008; Sperner-Unterweger et al. 2014; Felger 2018; Leblhuber et al. 2018a, 2019).

Repetitive transcranial magnetic stimulation (rTMS) is a noninvasive technique for stimulation of the human brain. Focused magnetic field pulses can influence brain function if delivered repetitively (Hallet 2007). Magnetic fields induced by rTMS can excite or inhibit a small brain area, thus altering cortical excitability (Hallet 2007; Noda et al. 2015). rTMS is described as a promising alternative strategy for treatment of resistant depression (Noda et al. 2015). rTMS was described earlier as effective intervention in depressive illness as augmentation in treatment-resistant cases (Liu et al. 2014, 2017). Further on, Iimori and colleagues (Iimori et al. 2019) could exert pro-cognitive effects of rTMS on executive function and attention in some patients with depression. Contrary to electroconvulsive therapy (ECT), rTMS does not provoke epileptic seizures and is associated with minimal side effects only (Koren et al. 2001).

rTMS over the prefrontal cortex is primarily targeting the processing of emotion and mood (Liu et al. 2017), but may also influence wide-ranging cortical–subcortical anatomical connectional systems including the thalamus important in perception, cognition and motivation (Pessoa, 2017). The medial frontopolar cortex [mFPC, Brodmann area 10 (BA 10)] and the amygdala play a major role in emotion and in mental disorders, probably relevant for novel treatment approaches in psychiatry (Riedel et al. 2019). Neuroimaging studies have shown that patients with major depressive disorder have dysfunctions in BA 10 (Katayama et al. 2019). New treatments increasing the activity and functional connectivity of the medial orbitofrontal cortex may be useful for treatment of depression (Rolls et al. 2020). Therefore, the mFPC was selected as rTMS target.

In this exploratory intervention study, the effect of rTMS on serum concentrations of neurotransmitters, BDNF, nitrite, inflammation markers and salivary amylase in patients with treatment-resistant depression were investigated: all of them were described in numerous earlier papers to be important in depressive syndromes. Compared to our earlier pilot studies (Leblhuber et al. 2018a, 2019) in which an influence of rTMS on phenylalanine levels had been observed, patients were exposed to a magnetic field that was approximately 20 times stronger than in the foregoing study (1.5 compared to 0.08 Tesla).

## Patients and methods

Thirty-eight consecutive patients with symptoms of treatment-resistant unipolar depression [incomplete remission of depressive symptoms after adequate antidepressant treatment (Thase 2011)] were included in this study (Table 1). On 10 subsequent working days, they underwent 10 active or sham rTMS stimulations of the mFPC with a TAMAS^®^ apparatus (DROTT Medizintechnik, Wiener Neudorf, Austria; Frequency 20 Hz, 1.5 Tesla, 2 s on, 28 s off, duration of treatment 30 min, 2400 stimuli per session). Every second outpatient was directed blindly to rTMS or sham treatment. To blind rTMS performer was not possible, because stimulation of the mFPC elicited visible bilateral contractions of the orbital musculature in every patient of the verum group (Vamava et al. 2011; Badran et al. 2019). Acoustic signal was identical for both the groups.

**Table 1:** Demographics (mean + SD) of the patients with depression treated with repetitive transcranial magnetic stimulation (*n* = 21) or SHAM (*n* = 17)

Depression episodes						
	Age (years)	Sex	Current (months)	Previous (*n*)	Comorbidity	Start
rTMS treated						
*n* = 21	59.4 ± 15.7	10 m, 11 f	3.6 ± 2.7	3.5 ± 1.5	5 hypertension	17 SSNRI
					4 DM	9 SSRI
					3 obesity	8 SARI
						5 antiepileptic
						4 NaSSA
						4 atyp.NL
						2 TCA
SHAM treated						
*n* = 17	61.2 ± 15.0	9 m, 8 f	4.2 ± 1.6	2.8 ± 1.4	5 hypertension	12 SARI
					4 obesity	10 SSNRI
					2 DM	5 SSRI
					2 Hyperchol	5 atyp.NL
					1 Hypothyr	5 NaSSA
					1 alcohol abuse	4 antiepileptic
						2 TCA

*Atyp. NL* atypical neuroleptic drug, *DM* diabetes mellitus, *Hyperchol* hypercholesterolemia, *Hypothyr* Hypothyreosis, *NaSSA* noradrenergic and specific serotonergic antidepressant, *SARI* serotonin antagonist and reuptake inhibitor, *SSNRI* selective serotonin/noradrenaline reuptake inhibitor, *SSRI* selective serotonin reuptake inhibitor, *TCA* tricyclic antidepressant

All patients were investigated as described earlier including routine laboratory tests and cerebral magnetic resonance tomography (MRT) to exclude circumscript cerebral lesions (Leblhuber et al. 2018a, b, 2019). Clinical assessment was performed within hours or within 1 day maximum using the 7-item Hamilton depression scale (HAMD-7; McIntyre et al. 2005) blindly to the treatment group allocation (KS). There was no difference in the medication of the verum and the sham group. SSRI and SSNRI medication was given in most patients, and was identical in both the verum and the sham group also consistently throughout rTMS treatment (Table 1). Patients included did not receive any psychotherapy during this intervention.

Blood was drawn always before noon, approximately at 10 a.m. The following parameters were controlled before and after rTMS treatment: serum concentrations of neopterin, tryptophan and kynurenine, calculating the kynurenine to tryptophan ratio (Kyn/Trp) as an index of tryptophan breakdown were determined with reverse phase HPLC as described previously (Widner et al. 1997; Leblhuber et al. 2019). As well phenylalanine and tyrosine concentrations were measured with HPLC applying slightly different conditions, and the phenylalanine to tyrosine ratio (Phe/Tyr) was calculated, serving as an index of phenylalanine hydroxylase (PAH) activity (Neurauter et al. 2013). In addition, serum concentrations of brain-derived neurotrophic factor (BDNF)—apparently mediating therapeutic benefits of rTMS (Niimi et al. 2016)—and of amylase in saliva were measured with commercially available ELISAs (BDNF: human-free BDNF Quantikine ELISA kit obtained from Biomedica, Vienna, Austria; α-amylase: Novus human salivary amylase alpha Elisa kit obtained from Tecan, IBL, Hamburg, Germany). Measurements were not performed in a fasted state. Serum samples were stored immediately at − 20 °C for later analysis, measurements were performed after study finish in one single run for each analyte.

Data were analyzed applying the Statistical Package for Social Science (version 19, SPSS, Chicago, IL, USA) as described earlier (Leblhuber et al. 2019). To take into account that not all collected data followed a normal distribution, non-parametric Friedman and Wilcoxon signed-rank tests were applied. To test for associations between variables, Spearman rank correlation analysis was performed, and *p* values below 0.05 were considered to indicate significance. ANOVA with repeated measurements was applied, as between-grouping factors “group” VERUM (patients) vs. SHAM (controls) were chosen, as within-grouping factor “phase” time points before and after treatment. In each ANOVA, number of observations was 38. For the overall models, there were 30 degrees of freedom, which were divided into 1 degree of freedom each for “treatment”, “time” and the interaction term “time#treatment”; the remaining 27 degrees of freedom being associated with the residual error. Calculations were performed with statistical package “STATA”, version 14.2 (StataCorp LLC, 4905 Lakeway Drive, College Station, Texas 77845, USA). *p* values below 0.05 were considered to indicate significance.

The study was approved by the local ethics committee. Patients were treated with rTMS after informed consent according the Declaration of Helsinki.

## Results

**Fig. 1:**
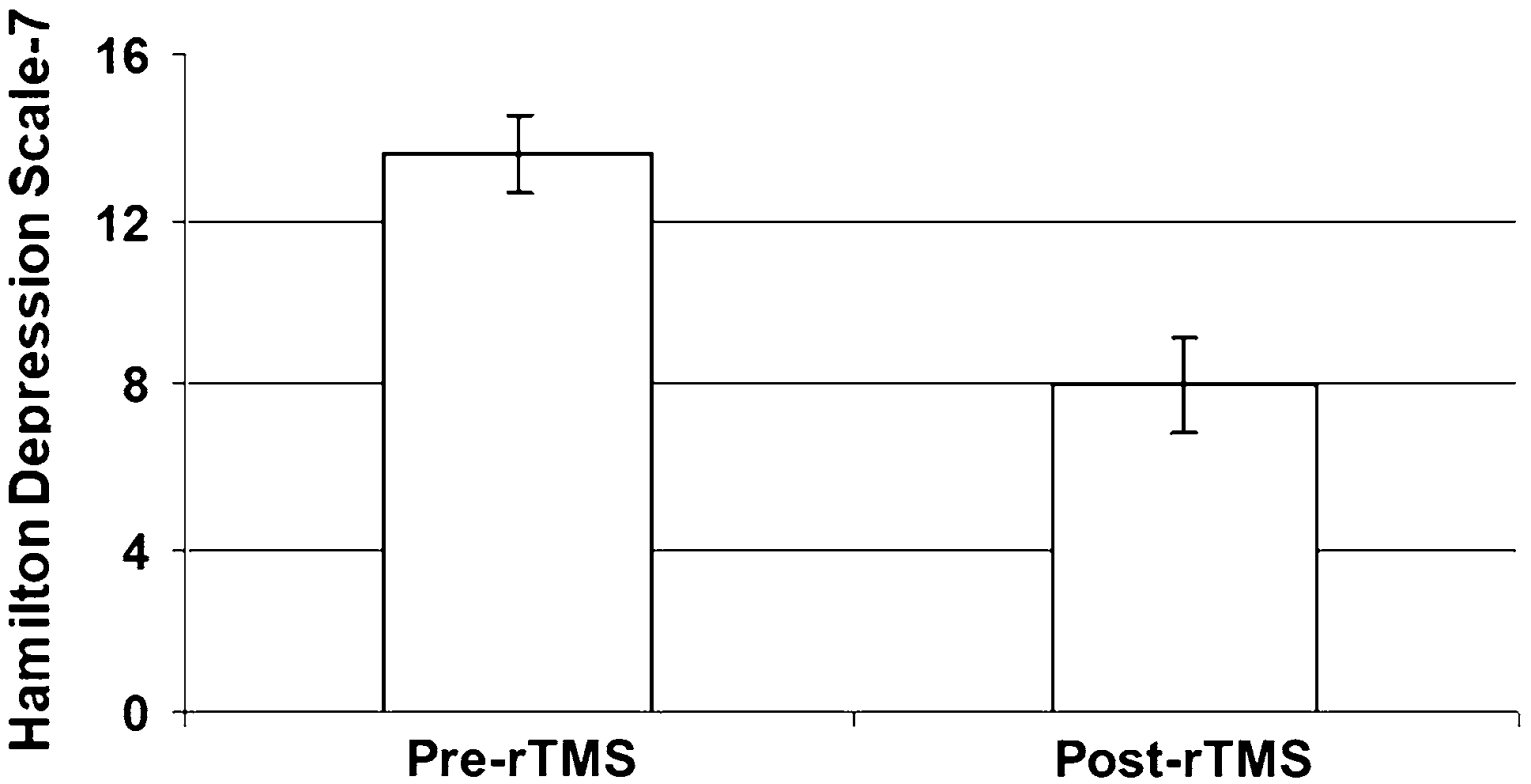
Influence of repetitive transcranial magnetic stimulation (rTMS) on depression intensity according to the HAMD-7 depression scale (*z* = 3.842, *p* = 0.001; mean values ± SEM are shown)

Routine whole blood laboratory tests including leucocyte count and C-reactive protein showed results within normal limits. In one of the cases, active treatment had to be discontinued due to “inconvenience” during the second rTMS session, feeling unpleasant to sit quiet for half an hour, but did not claim pain or other adverse symptoms. In all of the other cases intensity of rTMS was well tolerated. No serious adverse event was observed in any of the patients treated. rTMS induced significant depression symptom improvement and a significant decrease in the HAMD-7 score after active treatment (mean ± SEM, before 13.6 ± 0.96, after: 8.0 ± 1.09; z = 3.842, *p* = 0.001, Fig. 1). No effect was found in the SHAM-treated group (11.4 ± 1.23 before, after: 11.5 ± 1.01; *U* = 1.03, n.s.).

Serum concentrations of neurotransmitter precursor amino acids, neopterin, nitrite and BDNF before and after rTMS treatment are shown in Table 2.

**Table 2:** Serum concentrations (mean ± SEM) of neurotransmitter precursor amino acids, neopterin, nitrite and brain-derived neurotrophic factor (BDNF) in 21 patients with treatment-resistant depression (*Kyn/Trp* Kynurenine/Tryptophan ratio, *Phe/Tyr* phenylalanine/tyrosine ratio) before and after rTMS treatment sessions (*n.s.* not significant)

	Before rTMS				After rTMS				Wilcoxon test	
	Median	Mean	SD	SEM	Median	Mean	SD	SEM	*z*	*p*
Tryptophan (μmol/L)	54.7	57.7	11.9	2.65	50.3	54.1	11.3	2.53	1.373	n.s
Kynurenine (μmol/L)	1.99	1.93	0.62	0.14	1.76	1.99	0.72	0.16	0.400	n.s
K/T*1000 (μmol/mmol)	32.0	34.6	13.3	2.98	36.2	37.2	12.7	2.84	1.808	0.071
Tyrosine (μmol/L)	66.4	75.4	19.3	4.31	60.8	66.2	17.2	3.85	2.207	0.027
Phenylalanine (μmol/L)	76.5	83.8	21.1	4.71	67.9	71.9	16.2	3.63	2.172	0.030
Phe/Tyr (μmol/μmol)	1.09	1.12	0.16	0.04	1.06	1.11	0.19	0.04	0.560	n.s
Neopterin (nmol/L)	5.54	6.95	3.58	0.80	4.91	6.55	3.32	0.74	0.604	n.s
Nitrite (μmol/L)	13.2	21.0	20.1	4.50	10.7	15.8	16.7	3.74	0.317	n.s
BDNF (pg/ml)	147	161	104	22.2	147	136	112	24.0	0.330	n.s

**Fig. 2:**
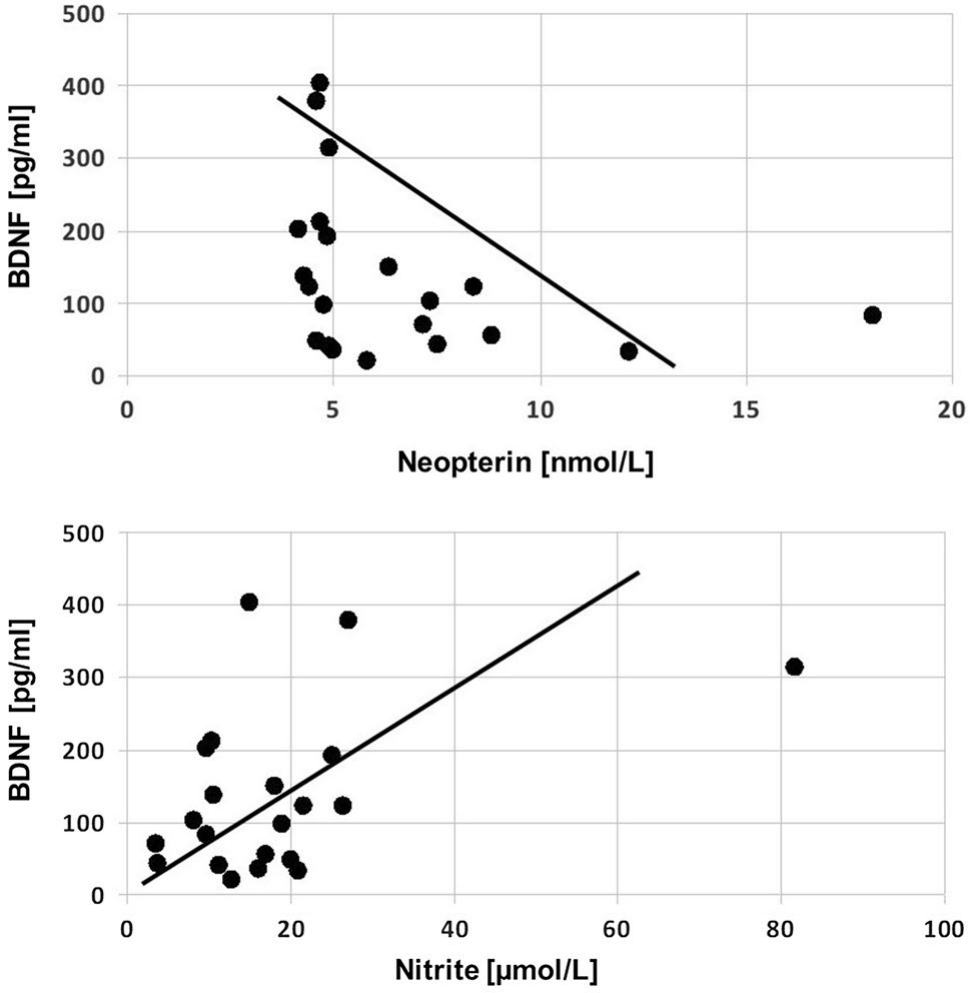
Associations between BDNF concentrations and neopterin (upper; *r*_*s*_ = − 0.528, *p* = 0.01) as well as nitrite (lower; *r*_*s*_ = 0.551, *p* < 0.01) concentrations in patients after repetitive transcranial magnetic stimulation (rTMS) suffering from depressionV

After rTMS, BDNF levels correlated inversely with serum neopterin (*r*_*s*_ = − 0.528, *p* = 0.01) and positively with nitrite (*r*_*s*_ = 0.551, *p* = 0.01; Fig. 2). Neopterin before rTMS correlated also inversely with BDNF levels after rTMS (*r*_*s*_ = − 0.436, *p* < 0.05). Furthermore, significant correlations existed between serum concentrations of neopterin and Kyn/Trp (*r*_*s*_ = 0.636, *p* < 0.001) before rTMS. After rTMS, this association was much less pronounced and failed to reach the level of significance (*r*_*s*_ = 0.424, *p* < 0.06).

Upon rTMS treatment, phenylalanine concentrations decreased significantly (*p* = 0.03) as well as tyrosine (*p* = 0.027) while Kyn/Trp tended to increase (*p* = 0.071, see Table 2). Salivary amylase concentrations neither correlated significantly with depression scores nor with any other biomarkers in this study and were not influenced by rTMS. No other changes of serum biomarkers were seen neither in patients nor in controls.

ANOVA revealed a significant effect only of phenylalanine for time (*p* = 0.027). The interaction term was insignificant and no other soluble marker concentrations showed significant influence.

## Discussion

The treatment of depression remains a challenge since approximately half of the patients do not respond to initial antidepressant therapy and 20% present with chronic symptoms for more than 2 years despite correctly administered standard treatment (Fava, 2003). Available treatments are ineffective in nearly 30–50% of patients, which may reflect a lack of specificity (Hodes et al. 2015). The underlying neurobiological determinants remain largely undefined (Duman et al. 2019), and new fast-onset antidepressant candidate targets besides the classical monoamine strategy have been proposed recently (Li 2020).

Significant alterations of neurotransmitter levels were previously reported in depressive syndromes (Price et al. 2009). Accumulating data suggesting an important role of the immune system in the development of mood disorders (Dantzer 2002; Widner et al. 2002) has been arising from the “sickness behavior” observed in inflammatory states. The predominant pro-inflammatory cytokines that cause behavioral signs of sickness are interleukin-1ß and tumor necrosis factor-α (Benedetti et al. 2020). Of interest, an influence of rTMS on these pro-inflammatory cytokines was described recently (Zhao et al. 2019).

The pro-inflammatory cytokine interferon-γ stimulates the biosynthesis of BH4, a cofactor for several mono-oxygenases which are rate limiting in the biosynthesis of serotonin, tyrosine and dopamine, and also of adrenaline and noradrenaline. Interferon-γ further induces and enhances the formation of reactive oxygen species (ROS) by macrophages (Nathan et al. 1983; Widner et al. 2000). ROS can then destroy the oxidation-labile BH4 and thus may thereby reduce the production of dopamine and catecholamines, all of them important in depression (Neurauter et al. 2008; Sperner-Unterweger et al. 2014).

BDNF concentrations correlated inversely with concentrations of neopterin before and after rTMS therapy. After the treatment sessions, nitrite levels correlated positively with BDNF levels. Nitrite levels may increase due to increased nitric oxide formation, and thus BDNF production might be associated with nitric oxide synthase activity. The inverse correlation of BDNF with neopterin levels further supports an association of BH4 biochemistry with BDNF production. Together, the associations found between BDNF and nitrite and neopterin levels (inverse) point to an impact of immunoregulatory circuits on BDNF levels, and this might relate to the beneficial effects of rTMS on depressive mood. The inverse correlation between BDNF and neopterin concentrations might indicate an influence of immune activation to suppress BDNF formation. However, the fact that BDNF derives from brain and BDNF and neopterin measurements were performed in serum only renders such an interpretation rather far reaching. Likewise, the positive association observed between serum levels of BDNF and nitrite as a surrogate for nitric oxide would make quite some sense but suffers from the same limitation.

rTMS is an evidence-based effective noninvasive treatment for major depressive disorder (Liu et al. 2014, 2017) approved by the US Food and Drug Administration (Connolly et al. 2012). This exploratory intervention study further describes, similar to an earlier series (Leblhuber et al. 2019) evidence regarding the safety and efficacy of rTMS in patients with treatment refractory depression. Patients were treated at ten consecutive working days with rTMS: rTMS induced a significant depression symptom improvement with a significant decrease in the HAMD-7 scale compared to sham-treated patients. Phenylalanine concentrations simultaneously declined similar to earlier findings in patients with severe depression responding to electroconvulsive therapy. Results further suggest a central role of BH4 activity in the pathophysiology of depression as already proposed by Anderson and coworkers in 1994 (Anderson et al. 1994). In fact, our data indicate that rTMS influences the PAH enzyme. PAH plays a key role in the biosynthesis of neurotransmitters dopamine, noradrenaline and adrenaline, all of which are downstream products of tyrosine. Decrease of tyrosine after rTMS application supports the possibility that this amino acid is used for synthesis of new neurotransmitters. Laboratory parameters were not measured in a fasted state. As already argued in the foregoing study (Leblhuber et al. 2019), the decrease of phenylalanine could possibly relate to changes in nutritional behavior of our patients studied, but concentrations of tryptophan—another essential amino acid—contrary to a recent series after hemispheric stimulation (Niimi et al. 2020) did not change significantly.

In addition, in the present series, a significant decline of tyrosine concentrations was found in parallel (*p* = 0.027), probably due to the higher power of the magnetic field approximately 20 times stronger than in the foregoing study, 1.5 compared to 0.08 Tesla (Leblhuber et al. 2019). Nevertheless, it is still unclear by which mechanism rTMS exactly contributes to the decrease of the concentrations of the neurotransmitter precursors. We suppose rTMS is effective by exerting a positive influence on PAH and downstream enzymes (Leblhuber et al. 2019). Interestingly, the decline of concentrations of the amino acids phenylalanine and tyrosine occurred rather independent of any noticeable change of biomarkers of immune activation, as concentrations of neopterin remained unchanged and Kyn/Trp tended to be higher after rTMS. Higher Kyn/Tryp is representative for tryptophan degradation mostly due to indoleamine 2,3-dioxygenase (IDO) activity, the enzyme being inducible by pro-inflammatory stimuli like interferon-γ thus indicating immune system activation. At the same time, IDO is involved in down-regulation of immune activation in a kind of inducing immune tolerance. It could thus represent an immunosuppressive strategy of the immune system to dampen the chronic immune response.

Results of our exploratory pilot study are promising, even if data from only 10 subsequent rTMS stimulations—due to personal and time burden—of 21 patients with medication resistant depression were included for verum rTMS treatment and 17 for sham treatment. We cannot extrapolate from our data how rTMS exactly influences neurotransmitter metabolism, and whether it may be a causal or rather symptomatic therapy. Immune activation does not seem be altered significantly by rTMS, rather stimulation of certain brain areas (increased functional connectivity of mFPC-BA10) and altered neurotransmitter production there might be affected. Further studies investigating biochemical pathways and specific brain areas in more detail might provide interesting new data (Riedel et al. 2019; Katayama et al. 2019; Rolls et al. 2020). However, the so far already existing information on rTMS in patients with depression might offer a potential new treatment option.

Of interest, intermittent Theta Burst Stimulation (iTBS), which has been used recently in clinical practice, could have a faster and more intense effect with less time burden in patients with treatment-resistant depression compared to conventional protocols (Bakker et al. 2015; Blumberger et al. 2018).
